# Preparation, Antioxidant Activities and Bioactive Components of Kombucha Beverages from Golden-Flower Tea (*Camellia petelotii)* and Honeysuckle-Flower Tea (*Lonicera japonica*)

**DOI:** 10.3390/foods12163010

**Published:** 2023-08-10

**Authors:** Si-Xia Wu, Ruo-Gu Xiong, Jin Cheng, Xiao-Yu Xu, Guo-Yi Tang, Si-Yu Huang, Dan-Dan Zhou, Adila Saimaiti, Ren-You Gan, Hua-Bin Li

**Affiliations:** 1 Guangdong Provincial Key Laboratory of Food, Nutrition and Health, Department of Nutrition, School of Public Health, Sun Yat-sen University, Guangzhou 510080, China; wusx6@mail2.sysu.edu.cn (S.-X.W.); xiongrg@mail2.sysu.edu.cn (R.-G.X.); chengj225@mail2.sysu.edu.cn (J.C.); huangsy9@mail2.sysu.edu.cn (S.-Y.H.); zhoudd6@mail2.sysu.edu.cn (D.-D.Z.); saimaiti@mail2.sysu.edu.cn (A.S.); 2 School of Chinese Medicine, Li Ka Shing Faculty of Medicine, The University of Hong Kong, Hong Kong 999077, China; u3008268@connect.hku.hk (X.-Y.X.); tanggy@connect.hku.hk (G.-Y.T.); 3 Singapore Institute of Food and Biotechnology Innovation (SIFBI), Agency for Science, Technology and Research (A*STAR), 31 Biopolis Way, Singapore 138669, Singapore

**Keywords:** kombucha, golden-flower tea, honeysuckle-flower tea, fermentation, antioxidant activities, polyphenols

## Abstract

Kombucha is a fermented tea known for its health benefits. In this study, golden-flower tea (*Camellia petelotii)* and honeysuckle-flower tea (*Lonicera japonica*) were first used as raw materials to prepare kombucha beverages. The antioxidant activities, total phenolic contents, concentrations of bioactive components, and sensory scores of two kombucha beverages were assessed. Additionally, effects of fermentation with or without tea residues on kombucha beverages were compared. The results found that two kombucha beverages possessed strong antioxidant activities and high scores of sensory analysis. In addition, fermentation with golden-flower tea residues could remarkably enhance the antioxidant activity (maximum 2.83 times) and total phenolic contents (3.48 times), while fermentation with honeysuckle tea residues had a minor effect. Furthermore, concentrations of several bioactive compounds could be increased by fermentation with golden-flower tea residues, but fermentation with honeysuckle-flower tea residues had limited effects. Moreover, the fermentation with or without tea residues showed no significant difference on sensory scores of golden-flower tea kombucha and honeysuckle-flower tea kombucha, and golden-flower tea kombucha had higher sensory scores than honeysuckle-flower tea kombucha. Therefore, it might be a better strategy to produce golden-flower tea kombucha by fermentation with tea residues, while honeysuckle-flower tea kombucha could be prepared without tea residues.

## 1. Introduction

Kombucha, a popular beverage in the world, is traditionally fermented from sweetened black tea infusions with tea fungus [1,2]. Kombucha contains various bioactive compounds which endow it with many bioactivities, such as antioxidant, antimicrobial, anticancer, antidiabetic, immunomodulatory, antidotal, hepatoprotective and cardiovascular protective activities [2,3,4]. In recent years, using non-*Camellia sinensis* teas, such as leaves, fruits, herbs, spices, milk and food industry by-products, as feasible substrates to prepare kombucha beverages has attracted more and more attention from researchers [5,6]. Many studies have shown that kombucha beverages based on non-*Camellia sinensis* teas have strong biological capacities as well as health-promoting properties [5,7,8]. For example, kombucha fermentation of African mustard leaves could significantly increase total phenolic content and improve antioxidant activity, compared with unfermented leaves beverage [9]. In the streptozotocin-induced diabetic rat model, kombucha from snake fruit exerted an antidiabetic effect via decreasing fasting plasma glucose, improving oxidation stress statuses and lipid profiles, which was better than the kombucha from black tea [10]. In addition to the innovation in alternative raw materials, several studies have pointed out that the polyphenol contents and antioxidant activity of kombucha were markedly increased by fermentation with tea residue [11,12].

Golden-flower tea (*Camellia petelotii*) and honeysuckle-flower tea (*Lonicera japonica*) are two famous edible and medicinal flowers [13,14]. Golden-flower tea contains various bioactive components, and has many health benefits, such as antioxidant, anticancer, antibacterial, hypoglycemic, hypolipidemic, immunomodulatory, antiallergic, anxiolytic and antidepressant effects [15,16,17,18,19]. Honeysuckle-flower tea contains a variety of bioactive compounds, and has been commonly used for the prevention and treatment of various diseases, because of its antiviral, anti-inflammatory, antitoxic, antioxidant, hypoglycemic, hypolipidemic, neuroprotective and immunomodulatory activities [20,21,22,23,24,25,26].

Although golden-flower tea and honeysuckle-flower tea have various bioactivities and health benefits, kombucha beverages from them have not been reported in the literature. In this study, kombucha beverages based on golden-flower tea and honeysuckle-flower tea were first studied through fermentation with or without tea residues. The antioxidant activities of these beverages were examined through the assay of ferric-reducing antioxidant power (FRAP) and Trolox equivalent antioxidant capacity (TEAC), and the total phenolic contents (TPC) were detected using the Folin–Ciocalteu method. Furthermore, certain bioactive compounds in these beverages were separated and determined using high-performance liquid chromatography with photodiode array detector (HPLC-PDA). Additionally, the sensory properties of these kombucha beverages were evaluated. Overall, these kombucha beverages have strong antioxidant activity and pleasant sensory properties.

## 2. Materials and Methods

### 2.1. Materials

The yellow dried tea from fresh golden-flower was produced in 2023 in Fangchenggang, Guangxi Province, China, and the green dried tea from fresh honeysuckle-flower was produced in 2023 in Fengqiu, Henan Province, China. The kombucha starter culture was obtained from Shandong Ruyun Edible Fungus Planting Co., Ltd. (Liaocheng, China).

Formic acid, sucrose and methanol were purchased from Macklin Chemical Factory (Shanghai, China). Sodium carbonate was purchased from Shanghai Yuanye Biological Technology Co., Ltd. (Shanghai, China). The 2,2′-azinobis (3-ethylbenothiazoline-6-sulfonic acid) diammonium salt (ABTS), 2,4,6-tri (2-pyridyl)-Striazine (TPTZ), 6-hydroxy-2,5,7,8-tetramethylchromane-2-carboxylic acid (Trolox), Folin–Ciocalteu’s phenol reagent and gallic acid were bought from Sigma-Aldrich (St. Louis, MO, USA). Hydrochloric acid, potassium peroxydisulfate, sodium acetate, iron(III) chloride hexahydrate, acetic acid and iron(II) sulfate heptahydrate were purchased from Tianjin Chemical Factory (Tianjin, China). The standard compounds ellagic acid, astragalin, rutin, chlorogenic acid, epicatechin, caffeine, quercitrin, gallic acid and quercetin were purchased from Derick Biotechnology Co., Ltd. (Chengdu, China). The standard phloretin was obtained from Ark Pharm, Inc. (Libertyville, IL, USA). Distilled water was used in this study.

### 2.2. Activation of Kombucha Starter Culture

The kombucha starter culture consists of tea fungus (a symbiotic culture of yeasts, acetic acid bacteria and lactic acid bacteria), fermented broth and cellulosic layer as well as teabag (5 g black tea). The kombucha starter culture was activated in accordance with the previous study with minor modifications [27]. At first, 100 g sucrose and 1 L water were boiled, and then 5 g black tea teabag was added. After infusing for 5 min, the teabag was carefully removed. The tea fungus, fermented broth and cellulosic layer were added after the tea infusion was cooled to room temperature (25 °C). The mixture was put in a clean and dark environment and fermented for 14 days at room temperature, which was used for subsequent experiments.

### 2.3. Kombucha Preparation Based on Golden-Flower Tea and Honeysuckle-Flower Tea

At first, 200 mL distilled water was added in each glass flask and heated in a boiling water bath. Successively, 20 g sucrose was added to each flask, and after the sucrose was dissolved in water completely, 2 g golden-flower tea or honeysuckle-flower tea was respectively added into the flasks and allowed to infuse for 5 min. After cooling to room temperature, the infusion was collected through filtering the mixture using a filter for fermentation without residues. In the case of fermentation with residues, the filtration step was skipped. Afterwards, 20 mL activated kombucha starter culture was added to each flask. Finally, four groups of kombucha drinks were obtained: (a) kombucha based on golden-flower tea without residues; (b) kombucha based on golden-flower tea with residues; (c) kombucha based on honeysuckle-flower tea without residues; (d) kombucha based on honeysuckle-flower tea with residues. Each group had 3 parallel samples. These conical flasks were put in a clean, dark and room-temperature environment and fermented for 18 days. On days 0, 3, 6, 9, 12, 15 and 18 of fermentation process, the samples were collected and filtrated by a 0.22 µm membrane for subsequent experiments.

### 2.4. Measurement of Antioxidant Capacity and TPC

The antioxidant activities of kombucha beverages were assessed by the FRAP and TEAC assays. The FRAP assay was conducted referring to a previous study [11], and the results were displayed as µmol Fe^2+^/L. Moreover, the TEAC assay was based on a previous study [12], and the results were expressed as µmol Trolox/L. In addition, the Folin–Ciocalteu method was carried out to evaluate the TPC according to a previous study [11], the results of which were in the form of mg of gallic acid equivalent (GAE)/L.

### 2.5. Determination of the Contents of Bioactive Components by HPLC-PDA

The contents of bioactive compounds in kombucha were determined by HPLC-PDA, following the previous literatures with minor adjustment [11,12,28]. An Agilent Zorbax Eclipse XDB-C18 column (Santa Clara, CA, USA) was used to separate, the size of which was 250 mm × 4.6 mm, 5 µm. The temperature of sample chamber and column was 4 °C and 35 °C, respectively. The mobile phase A was made up of methanol, and mobile phase B was 0.1% formic acid. The flow rate was 0.8 mL/min. The procedure of elution was the same as that in the literature [12].

### 2.6. Sensory Evaluation

The sensory property (color, odor, sourness, flavor and overall acceptability) of kombucha was evaluated according to previous studies [8,11,12,29]. Different types of kombucha samples were assessed by 8 participants, and the scoring criteria are the same as that in the literature [12]. The 8 participants come from the Department of Nutrition, School of Public Health, Sun Yat-sen University. They had good experience of sensory assessment and had participated in sensory evaluation in our previous studies [11,12].

### 2.7. Statistical Analysis

The statistical analysis was performed through the SPSS 25.0 statistical software (IBM Corp., Armonk, NY, USA) and Microsoft Excel 2021 (Washington, DC, USA). The one-way analysis of variance (ANOVA) followed by Tukey test was used to assess the statistical significance, and differences were considered significant at *p* < 0.05. Moreover, Pearson correlation index was used to analyze the correlations between parameters and compound concentrations. Additionally, the heatmaps were performed (https://www.chiplot.online accessed on 19 May 2023). All experimental results were represented as mean values and standard deviation.

## 3. Results and Discussion

**Figure 1:**
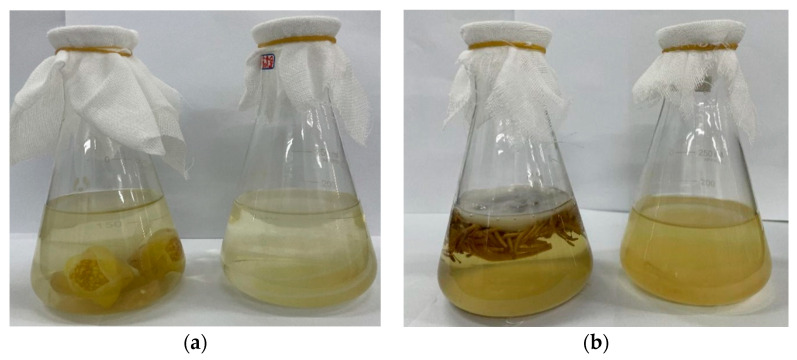
The appearance of kombucha based on golden-flower tea and honeysuckle-flower tea. (**a**) Kombucha based on golden-flower tea with or without residues; (**b**) Kombucha based on honeysuckle-flower tea with or without residues.

In this study, golden-flower tea and honeysuckle-flower tea were used as materials to prepare new kombucha beverages. The appearance of kombucha based on golden-flower tea and honeysuckle-flower tea are shown in Figure 1.

### 3.1. FRAP Values

**Figure 2:**
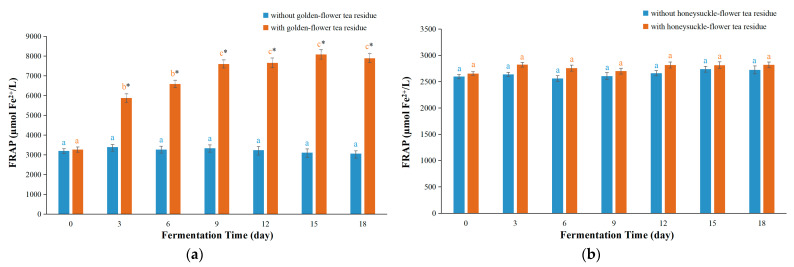
The FRAP values of kombucha beverages during fermentation process. (**a**) Kombucha based on golden-flower tea; (**b**) Kombucha based on honeysuckle-flower tea. In the same type of kombucha, different letters indicate significant differences (*p* < 0.05). * Denotes a significant difference (*p* < 0.05) between fermentation with residues and those without residues.

The FRAP assay could assess the reducing ability of substances on ferric ions [30]. For kombucha based on golden-flower tea with residues, the FRAP values firstly raised and reached its maximum value on day 15, then reduced slightly but not significantly on day 18 (Figure 2a). For kombucha based on golden-flower tea without residues, the FRAP values varied slightly in the first 9 days and then started to decrease slowly. Notably, the FRAP values of kombucha based on golden-flower tea with residues were remarkably higher (maximum 2.6 times) than those without residues. In the literature, several studies also reported that fermentation with residues might increase the FRAP values in kombucha compared with those without residues, such as black tea residues (1.6 folds), green tea residues (3.13 folds), vine tea residues (1.09 folds) or sweet tea residues (1.13 folds) [11,12], which indicated that raw materials could be used more effectively in fermentation with residues. For kombucha based on honeysuckle-flower tea, the FRAP values in honeysuckle-flower tea kombucha with or without residues changed little (Figure 2b). Additionally, the differences in the FRAP values between honeysuckle-flower tea kombucha with residues and those without residues were not statistically significant. These results indicated that fermentation with honeysuckle-flower tea residues had little effect on the FRAP values of the beverage, which were very different from fermentation with golden-flower tea residues.

### 3.2. TEAC Values

**Figure 3:**
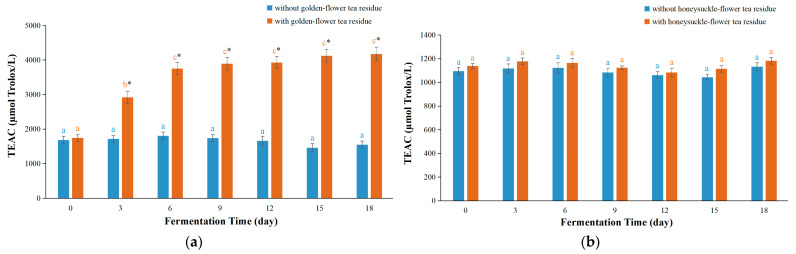
The TEAC values of kombucha beverages during fermentation process. (**a**) Kombucha based on golden-flower tea; (**b**) Kombucha based on honeysuckle-flower tea. In the same type of kombucha, different letters indicate significant differences (*p* < 0.05). * Denotes a significant difference (*p* < 0.05) between fermentation with residues and those without residues.

The antioxidant activity of substances are evaluated by the TEAC assay through comparing their ability for scavenging ABTS^•+^ radical cations [31]. For golden-flower tea kombucha with residues, the TEAC values increased rapidly until day 6 and then almost kept constant (Figure 3a). The maximum TEAC value was 4174.3 ± 197.1 µmol Trolox/L on day 18. For kombucha based on golden-flower tea without residues, the TEAC values changed little in the first 6 days, after which there was a slight decrease, but these changes were not significant. Prominently, the TEAC values of kombucha based on golden-flower tea with residues were obviously higher than those without residues (maximum 2.83 times). This effect was also found in kombucha based on black tea residues, sweet tea residues, vine tea residues, and green tea residues (1.61, 1.38, 1.3 and 3.25 folds, respectively) as reported in the previous studies [11,12], which indicated that these plant materials could be used more effectively through fermentation with tea residues.

For kombucha based on honeysuckle-flower tea, the TEAC values changed little in with or without residues (Figure 3b). Furthermore, no statistically significant difference was revealed in the TEAC values between honeysuckle-flower tea kombucha with residues and those without residues during fermentation process. These results showed that fermentation with honeysuckle-flower tea residues had little effect on the TEAC values of the beverage, which were consistent with the FRAP results above mentioned.

### 3.3. TPC Values

**Figure 4:**
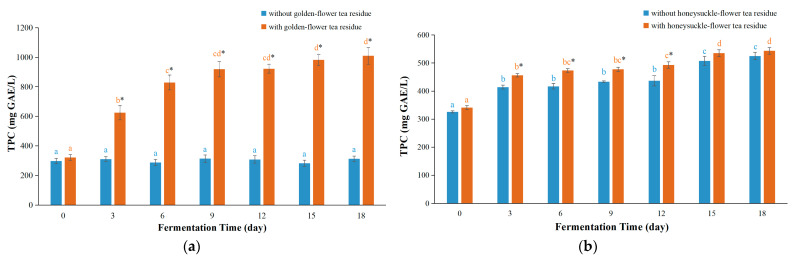
The TPC values of kombucha beverages during fermentation process. (**a**) Kombucha based on golden-flower tea; (**b**) Kombucha based on honeysuckle-flower tea. In the same type of kombucha, different letters indicate significant differences (*p* < 0.05). * Denotes a significant difference (*p* < 0.05) between fermentation with residues and those without residues.

The Folin–Ciocalteu method depends on reducing the capacity of phenols on Folin–Ciocalteu reagent using gallic acid as the standard to detect the TPC of plant samples or food products, which has been widely applied in a large amount of studies [11,12,32]. The TPC values in kombucha based on golden-flower tea with residues increased rapidly until day 9 and slowly thereafter (Figure 4a). The maximum TPC values were 1009.5 ± 56.9 mg GAE/L on day 18. For kombucha based on golden-flower tea without residues, the TPC values changed little. Moreover, the TPC values in kombucha with residues were found to be up to 3.48 times higher than those without residues. This implied that the presence of tea residues played a crucial role in enhancing the TPC in kombucha. The reason might be that one single extraction of boiling water was tough to extract all bioactive compounds of tea leaves [33,34], so certain bioactive compounds would remain in the leaves. During the fermentation process, these bioactive components could be dissolved by the action of enzymes, leading to elevated TPC values in kombucha based on golden-flower tea with residues. This fold was also remarkably higher than those of kombucha based on black tea residues (1.62 folds), green tea residues (2.98 folds), vine tea residues (1.55 folds), or sweet tea residues (1.35 folds) in the literature [11,12]. Furthermore, the TPC values of kombucha based on golden-flower tea with residues in this study was found to be markedly higher than those of another study where kombucha was prepared using black tea (412.25 ± 3.86 mg GAE/L), whereas the TPC values without residues were slightly lower [29]. Therefore, utilizing golden-flower tea residues for the preparation of kombucha could increase levels of total phenolics, thereby enhancing its nutritional value and potential health benefits of kombucha.

The TPC values of kombucha based on honeysuckle-flower tea fermented with or without residues are shown in Figure 4b, and both of them slowly increased. Moreover, the TPC values in kombucha based on honeysuckle-flower tea with residues were as 1.14 times as those without residues on day 9, which were lower than those of kombucha based on golden-flower tea with residues in this study. Moreover, the TPC values of kombucha based on honeysuckle-flower tea with residues (543.45 ± 11.74 mg GAE/L) or without residues (525.16 ± 13.42 mg GAE/L) were slightly higher than those of kombucha based on black tea (412.25 ± 3.86 mg GAE/L) in a previous study [29]. This indicated that honeysuckle-flower tea as raw material could increase the kombucha TPC values.

### 3.4. Contents of Bioactive Components

**Figure 5:**
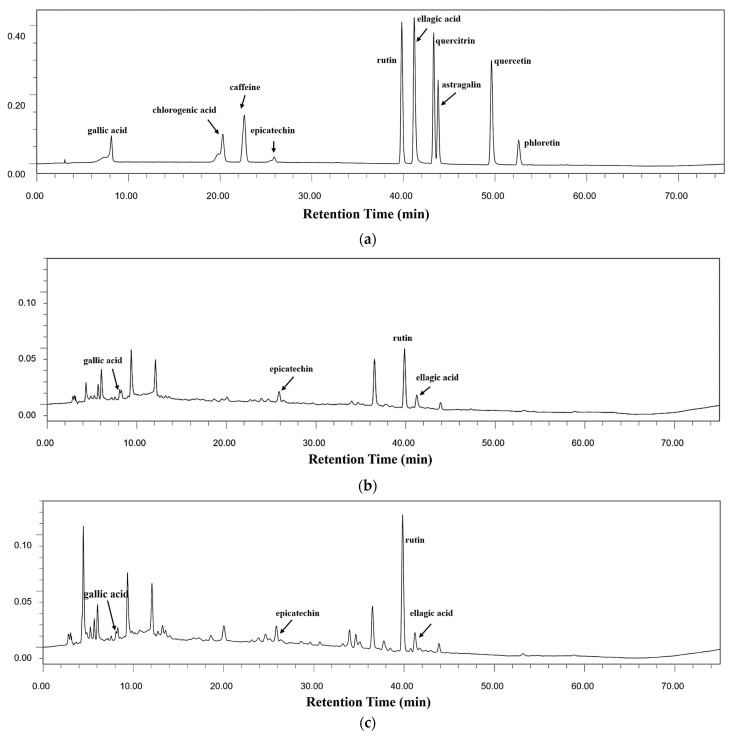

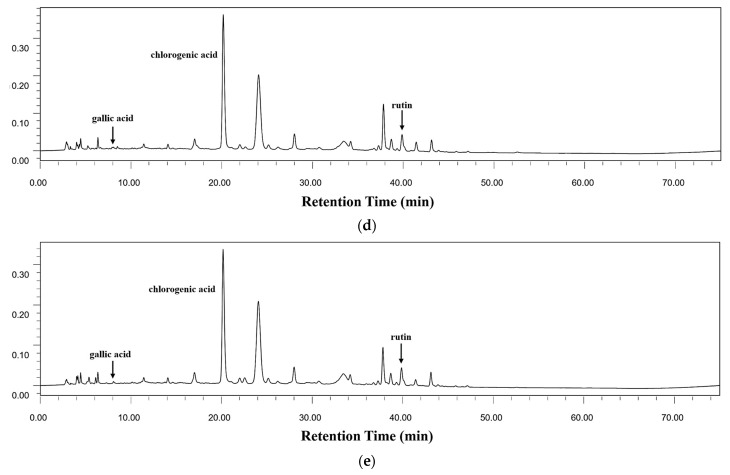
Chromatograms of standards and kombucha beverages at 254 nm. (**a**) Standards, (**b**) Kombucha based on golden-flower tea without tea residues, (**c**) Kombucha based on golden-flower tea with tea residues, (**d**) Kombucha based on honeysuckle-flower tea without tea residues and (**e**) Kombucha based on honeysuckle-flower tea with tea residues.

The bioactive compounds in kombucha were detached and determined, and their representative chromatograms are presented in Figure 5. Specifically, four main bioactive components (gallic acid, epicatechin, rutin and ellagic acid) were identified from kombucha based on golden-flower tea (Figure 5b,c). Furthermore, there were three main components (gallic acid, rutin and chlorogenic acid) identified from honeysuckle-flower tea kombucha (Figure 5d,e).

**Figure 6:**
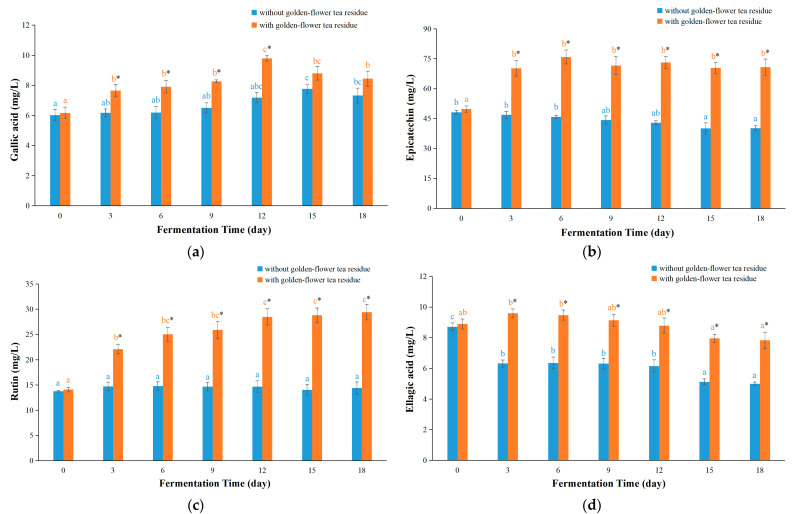

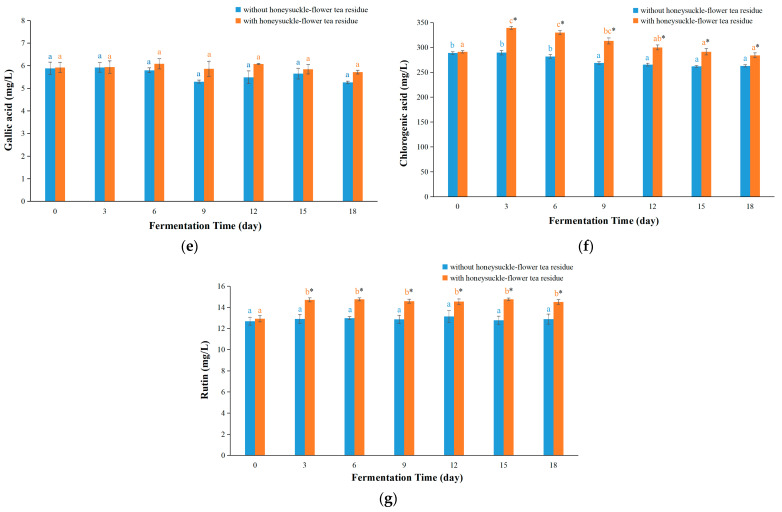
The contents of bioactive components in kombucha beverages at different fermentation time. (**a**–**d**) Kombucha based on golden-flower tea; (**e**–**g**) Kombucha based on honeysuckle-flower tea. In the same type of kombucha, different letters indicate significant differences (*p* < 0.05). * Denotes a significant difference (*p* < 0.05) between fermentation with residues and those without residues.

In general, the contents of the four compounds in kombucha based on golden-flower tea with residues were all higher than those without residues (Figure 6a–d). The reason might be the continuous dissolution of these components from the tea residues into the broth, similar to what is observed in kombucha based on green tea, black tea, vine tea or sweet tea [11,12]. Furthermore, the contents of epicatechin as well as ellagic acid in kombucha based on golden-flower tea with residues were firstly increased and then reduced (Figure 6b,d). It might be a result of complex interactions between extraction, degradation and microbial activity. The initial increase in the content of epicatechin and ellagic acid may be due to the extended extraction and the degradation of other compounds to produce epicatechin and ellagic acid [35]. At the same time, epicatechin and ellagic acid could be degraded by the action of microorganisms in kombucha broth [11]. In this study, the production of epicatechin and ellagic acid from other compounds was firstly higher than the degradation of epicatechin and ellagic acid by microorganisms and then was inverse. In contrast to previous studies on kombucha from green tea residues, black tea residues, sweet tea residues or vine tea residues [11,12], the concentration of gallic acid was firstly raised and then reduced in kombucha based on golden-flower tea with residues (Figure 6a). This might be because some compounds which could be degraded to produce gallic acid were lower in the golden-flower tea than in the black, green, vine and sweet tea. Additionally, the change trend of rutin concentration in kombucha with residues was raised (Figure 6c), which was different from that observed in kombucha based on sweet tea, where the content of rutin was first enhanced and then decreased in the fermentation progress [12]. The reason could be that the content of rutin in golden-flower tea was higher than in sweet tea, and the concentrations of rutin dissolved from golden-flower tea residue were higher than those degraded through microorganisms in the fermentation process.

For the kombucha from honeysuckle-flower tea, the content changes of the three compounds are shown in Figure 6e–g. In contrast to previous studies on kombucha fermented with green tea residues or black tea residues [11], the concentration of gallic acid in honeysuckle-flower tea kombucha almost did not vary (Figure 6e). This might be due to the fact that bioactive compounds in honeysuckle-flower tea are unlike those in black, green and white tea, and no related compounds in honeysuckle-flower tea might not be degraded to produce gallic acid [36]. Notably, kombucha from honeysuckle-flower tea had a very high concentration of chlorogenic acid, and the content of chlorogenic acid in kombucha with residues was higher than those of kombucha without residues since day 3 (Figure 6f). This could be because that chlorogenic acid could be continuously dissolved from honeysuckle-flower tea residues to the broth. In addition, the rutin content in kombucha based on honeysuckle-flower tea with residues was slightly increased at first and then almost unchanged. This could be because rutin was dissolved from honeysuckle-flower tea residues to the broth before day 3. On the other hand, rutin in kombucha without honeysuckle-flower tea residues did not change (Figure 6g).

### 3.5. Correlations Analysis between the Antioxidant Capacities and Bioactive Components

**Figure 7:**
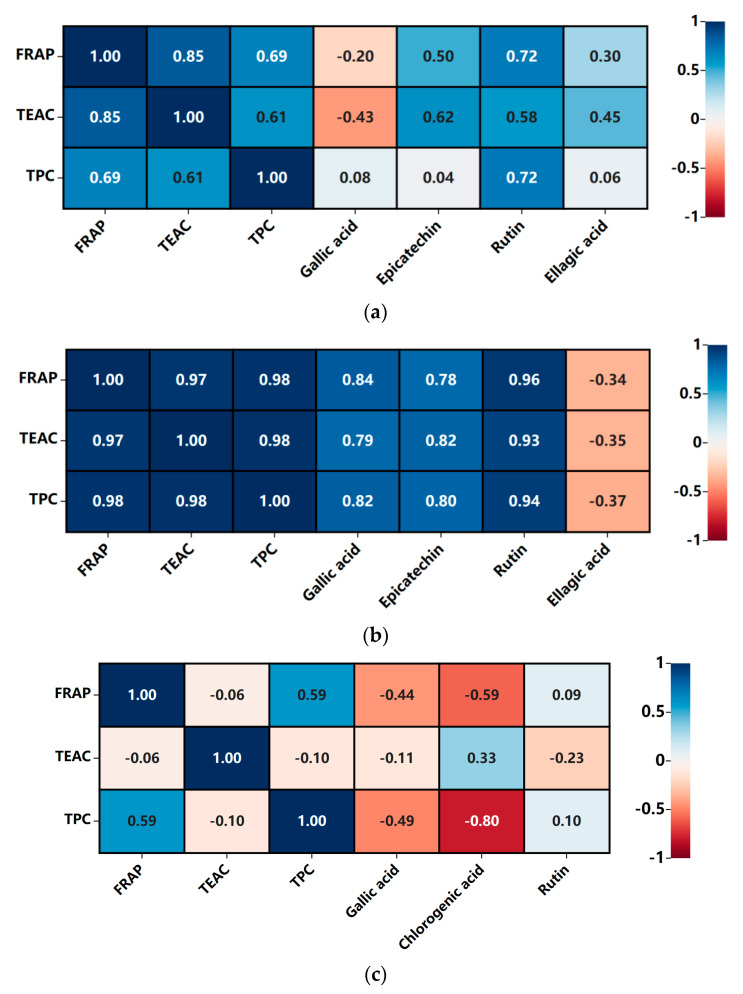

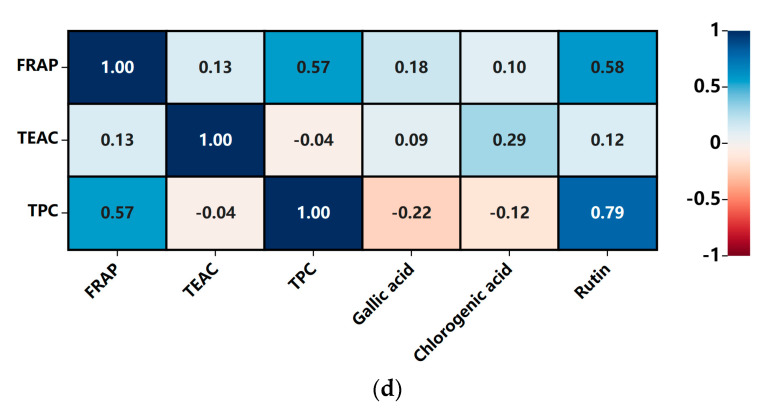
Heatmap analysis of compound concentrations and related parameters. (**a**) Kombucha based on golden-flower tea without residues, (**b**) Kombucha based on golden-flower tea with residues, (**c**) Kombucha based on honeysuckle-flower tea without residues and (**d**) Kombucha based on honeysuckle-flower tea with residues.

The correlation between FRAP, TEAC, TPC and contents of bioactive components was analyzed using the heat maps analysis (Figure 7).

For FRAP and TEAC values, they were remarkably correlated in golden-flower tea kombucha (without residues: R = 0.85; with residues: R = 0.97), while those in honeysuckle-flower tea kombucha were not significantly correlated. It suggested that antioxidant compounds in golden-flower tea kombucha but not honeysuckle-flower tea kombucha could possess multi-function to reduce oxidants (such as ferric ions) and scavenge free radicals [30]. Additionally, the correlation between FRAP and TEAC values in golden-flower tea kombucha with residues was stronger than in that without residues.

For FRAP and TPC values of golden-flower tea kombucha, significant correlations were found (without residues: R = 0.69; with residues: R = 0.98), while no significant correlations were shown in honeysuckle-flower tea kombucha. This indicated that phenolic components could have contributed to reducing the capacity of golden-flower tea kombucha.

For TEAC and TPC values of kombucha prepared from golden-flower tea, significant correlations were shown (without residues: R = 0.61; with residues: R = 0.98), but no significant correlations were found in kombucha prepared from honeysuckle-flower tea. This indicated that phenolic components could be the contributors of scavenging free radicals in golden-flower tea kombucha but not honeysuckle-flower tea kombucha.

With regards to FRAP values and bioactive components in golden-flower tea kombucha, (1) significant association existed between FRAP and contents of rutin in kombucha without residues (R = 0.72); and (2) FRAP values were significantly correlated with concentrations of gallic acid, epicatechin and rutin in kombucha with residues (R = 0.84, 0.78, 0.96, respectively). The results suggested that these compounds might play a role in the FRAP values of golden-flower tea kombucha. However, FRAP values were not significantly correlated with the detected bioactive components in honeysuckle-flower tea kombucha without residues or with residues.

With regards to TEAC values and bioactive components in golden-flower tea kombucha, (1) significant association was shown between TEAC and concentrations of epicatechin in kombucha without residues (R = 0.62); and (2) significant relationships were presented between TEAC and concentrations of gallic acid, epicatechin and rutin in kombucha with residues (R = 0.79, 0.82, 0.93, respectively). This indicated that these components might play a role in the TEAC values of golden-flower tea kombucha. However, TEAC values were not significant correlated with the detected bioactive components in honeysuckle-flower tea kombucha without residues or with residues.

As for correlations between TPC values and bioactive components in golden-flower tea kombucha, (1) significant association was found between TPC and concentrations of rutin in kombucha without residues (R = 0.72); and (2) TPC values were significantly correlated with concentrations of gallic acid, epicatechin and rutin in kombucha with residues (R = 0.82, 0.80, 0.94, respectively). This suggested that these components might be the contributors of the TPC values in golden-flower tea kombucha. Regarding the correlations between TPC values and bioactive components in honeysuckle-flower tea kombucha, a significant correlation was shown between TPC and concentrations of rutin in kombucha with residue (R = 0.79), which indicated that rutin might significantly contribute to the TPC values of the beverage.

Because free radicals/oxidative stress could cause several chronic diseases, these kombucha beverages with strong antioxidant capacities could be used for the prevention and management of oxidative stress-related diseases, such as cardiovascular diseases, diabetes mellitus and some cancers, which needs to be verified in the future studies.

### 3.6. Sensory Evaluation

**Figure 8:**
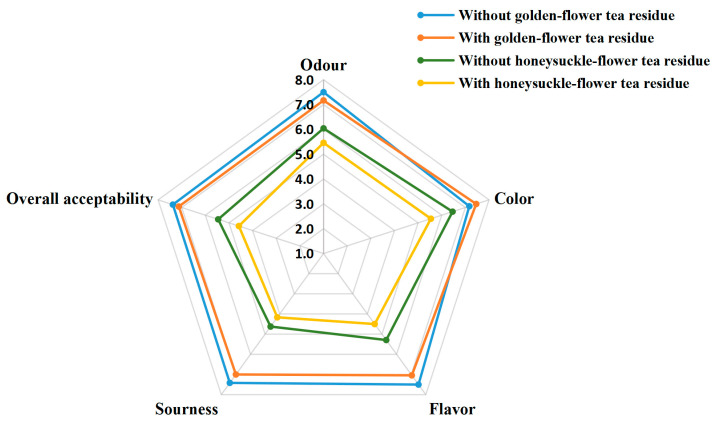
Results of sensory evaluation.

The results of sensory evaluation are presented in Figure 8. No significant differences in the scores of these five evaluated sensory parameters were observed between golden-flower tea kombucha with residues and those without residues. Moreover, there also were no significant differences in the scores between honeysuckle-flower tea kombucha with residues and those without residues. However, the kombucha based on golden-flower tea had markedly higher scores than those of kombucha based on honeysuckle-flower tea. Perhaps, golden-flower tea kombucha was a better beverage compared with honeysuckle-flower tea kombucha to prevent several oxidative stress-related diseases, because it also had higher TPC values and stronger antioxidant activities.

## 4. Conclusions and Perspectives

In this study, kombucha beverages prepared with golden-flower tea and honeysuckle-flower tea have been studied for the first time. The results found that these kombucha beverages all have high antioxidant capacities and TPC values, and fermentation with golden-flower tea residues could markedly increase the antioxidant activities and TPC values of kombucha beverages, while fermentation with honeysuckle-flower tea residues had little effect. Moreover, several bioactive components in these kombucha beverages have been separated and determined. In general, fermentation based on golden-flower tea with residues might remarkably enhance the contents of several bioactive compounds, and fermentation based on honeysuckle-flower tea with residues might slightly raise the contents of bioactive compounds. Additionally, sensory analysis showed that kombucha fermented with golden-flower tea had higher scores than that fermented with honeysuckle-flower tea. Thus, fermentation with golden-flower tea residues might be more suitable for the preparation of kombucha beverages due to its potent antioxidant activities and abundant phenolic contents, and honeysuckle-flower tea kombucha beverages could be prepared from fermentation without residues. These kombucha beverages could be used as functional food to prevent several oxidative stress-related diseases.

## Data Availability

Data are contained within the article.
